# Explanatory models of illicit drug use in adolescents: A qualitative study from India

**DOI:** 10.1371/journal.pgph.0003647

**Published:** 2024-10-14

**Authors:** Bijayalaxmi Biswal, Brian Zhou, Karen Wen, Devika Gupta, Urvita Bhatia, Abhijit Nadkarni

**Affiliations:** 1 Addictions and related-Research Group, Sangath, Goa, India; 2 Department of Anthropology, Harvard University, Cambridge, Massachusetts, United States of America; 3 Department of Public Health, London School of Hygiene and Tropical Medicine, London, United Kingdom; 4 Department of Population Health, London School of Hygiene and Tropical Medicine, London, United Kingdom; McGill University, CANADA

## Abstract

Illicit drug use is a growing concern in India, with a high treatment gap of 73%. Explanatory models can provide valuable insights into the patient’s conception of disease and inform help-seeking, treatment, and recovery. Of the studies that examine adolescent drug misuse in India, none have developed a socio-cultural explanatory model. The aim of our study was to develop an explanatory model to better understand the causal beliefs, social context, and self-perception of illicit drug misuse amongst adolescents in India. We conducted semi-structured interviews with 23 adolescents seeking treatment for drug use disorders and 25 healthcare providers recruited across three sites in India. Thematic analysis was used to analyse data. Most adolescent participants reported using multiple drugs, often in combination with cannabis. Frequent usage was reported i.e., daily at multiple times. Causes of initiation and continued use were peer influence, curiosity and pleasure, psychosocial stressors, family conditions, and systemic risk factors (e.g. socioeconomic instability). Drugs were acquired from various sources, including fellow users and pharmacies. Adolescent participants perceived negative impacts of drug use on physical and mental health, family relationships, and everyday functioning. Our findings on common reasons of drug use initiation, importance of peer relationships in continuation of use, impact of use on various aspects of life and the relationship of illicit drug use with socioeconomic status are consistent with previous research done in India on the subject. Understanding how adolescents and caregivers perceive drug use can help inform patient-clinician rapport, improve treatment compliance and understand intervention effectiveness. Such an explanatory model holds crucial implications for shaping interventions and clinical approaches to address adolescent drug use in India.

## Introduction

In 2019, 18.1 million Disability Adjusted Life Years (DALYs) and 0.7% of all DALYs globally were attributable to drug use. In the 10–24 years age group, 3.7 million DALYs and 1.6% of all DALYs were linked to drug use [1]. The burden ascribed to substance use increases substantially in adolescence and young adulthood [2], disrupting transitions to adulthood and entrenching substance use disorders [3].

In India, illicit drug use (use of illegal drugs) is a growing concern: the prevalence in some states is 25–40% with a treatment gap of 73% [4–6]. While addictions research in India has primarily focused on legal or “licit” substances like tobacco and alcohol, the limited evidence on illicit substances among adolescents indicates use of cannabis, opioids, and amphetamines [7–10]. One study showed that illicit substance use increased from 26% among 13-15-year-old adolescents to 44% in the 17–19-year age group [11].

The few studies that examine adolescent substance misuse in India have identified stress and pleasure-seeking as primary reasons for drug use, but a comprehensive sociocultural explanatory model exploring onset and progression, causal beliefs, and perceived impact of drug use is yet to be proposed. Global evidence suggests that the experience of adolescence, the cultural and legal responses to substance use, as well as causal attributions attached to drug use differ across cultural contexts [3]. Studies have also shown culturally specific variations in motivations, coping strategies, and barriers to help-seeking associated with substance use disorders [3, 12–21]. The wide-ranging reasons for drug use in these studies include: family instability and growing up in an addiction-affected family, lack of recreational avenues, self-medication for mental distress or physical pain, and curiosity or self-exploration [16–21]. Barriers to help-seeking differed across contexts, from fear of persecution or legal repercussions to feeling unworthy of treatment, a sense of hopelessness about recovery, and lack of information on available treatment options [16–21]. While these previous studies have quantitatively and separately assessed adolescent motivations for drug use, barriers and facilitators to help-seeking, and treatment ideals, we are the first to conduct a qualitative study synthesizing these distinct elements into a comprehensive sociogenic model of the experience and treatment of illicit drug use, from the perspectives of both adolescents and their health providers across multiple regions in India. The substantial variation in contextual manifestations of substance misuse necessitates a holistic explanatory model of disease that incorporates sociocultural aspects of drug use to transcend traditional biomedical models, which often reduce complex mental “illness” to purely neurological causes [22, 23].

The overall aim of our study was to develop an explanatory model for illicit drug use in adolescents to better understand the social context, causal beliefs, and perceived impact of illness within this key population in India. According to Kleinman, explanatory models describe aetiology, onset of symptoms, the disease process, course of illness, and treatment ideals [24]. By providing valuable insight into patients’ conception of disease, their wider belief systems, and social context, explanatory models can incorporate patients’ worldviews into culturally appropriate treatments [25].

In addition to exploring patients’ perceptions of their illnesses, we found it valuable to understand the physician-patient relationship and how health providers perceive drug use, and choose to treat it. By engaging with both help-seeking adolescents and healthcare providers, we aimed to gain deeper insights into the subjective experiences of everyone involved in the clinical course of treating illicit drug use [24].

In this paper, we report the first four aspects of adolescent drug use. Specifically, we seek to understand the characteristics (i.e., motivations, origins, and impact) of illicit drug use in adolescents by investigating the sociocultural attitudes, systems, and social determinants that contribute to drug use disorders. Coping strategies, barriers to help-seeking and desired treatment options are reported in a paired publication.

## Methods

### Study design

We used semi-structured in-depth interviews to acquire evidence from interviewees. This method ensures a confidential space for the participant to share their experiences, allows ample time for the interviewer to establish rapport given the sensitive nature of questions, and provides flexibility to explore topics that arise spontaneously [26, 27]. Interview questions and data analysis were operationalized under an ‘Illness Explanatory Model approach’ [24, 28]. Explanatory models (EMs) are defined as ‘notions about an episode of sickness and its treatment that are employed by all those engaged in the clinical process’ [24]. We adapted each of Kleinman’s Eight Questions (e.g. “What do you think has caused your problem?”) into open-ended formats for interviewing (e.g. “What factors in your life contribute to your usage?”) and themes for analysis (e.g. “causal beliefs”). Themes related to help-seeking and treatment are reported in another manuscript.

### Setting and sample

Adolescence spans from ages 10–24 years [29], and drug use peaks in those aged 18–25 years. We interviewed 18- to 24-year-old adolescents, who were currently in treatment for illicit drug use, and healthcare providers involved in delivering services to adolescents seeking help for substance use. Illicit drugs are defined as substances that are not medically prescribed and/or are illegal to consume, produce, and sell: psychedelic drugs (e.g. LSD); cannabis; inhalants (e.g. glue); opioids; sedatives (e.g. ketamine); and stimulants (e.g. amphetamines).

Because adolescents seeking help for illicit drug use are a hard-to-reach population, snowball sampling was used to identify adolescents. Purposive sampling was used to identify healthcare providers with experience of working closely with these adolescents. Participants were recruited from sites that vary geographically to capture regional variations in experiences (Delhi in North India, Goa in Southwest India, and Kerala in South India) between 1^st^ June and 31^st^ December 2019.

Adolescent participants were included if they were 18–24 years old, could either speak English or the local language (i.e languages understood by the research team/interpreters), were resident of India, and were seeking help for drug use. Healthcare providers were included if they had provided care to an adolescent seeking help for drug use, at least once in the past year.

### Data collection

We conducted semi-structured interviews of 60–75 mins each, with 23 adolescents seeking treatment for drug use disorders and 25 healthcare providers in total. Separate interview guides were used for substance users and health providers. A socio-demographic form recorded participant data on drug use, employment/income status, education, gender, location, and type of health centre. All participants were interviewed in-person at the health facility and the interviews were audio recorded. The interview guides were developed after reviewing literature on illicit drug use in India, ensuring that the questions were appropriate to guide inquiry into our study objectives. Preliminary analysis of initial interview data was conducted to add probes to the guides. The consent forms and socio-demographic forms were available in local languages and the semi-structured interview guide was in English.

A female researcher who had an undergraduate degree in psychology and training in qualitative interviewing (KW) led the interviews. 27 interviews were conducted in English, while 21 interviews were conducted in local languages with assistance from local interpreters from the health facilities who received basic training in qualitative interviewing. These interpreters helped resolve potential cultural differences in the comprehension of illness-related idioms between the non-native interviewer and the participants. The audio recordings were first transcribed and then translated into English by bilingual translators. Four of the interviews were not audio-recorded due to refusal of consent for recording; instead, the researcher wrote down participants’ responses.

The interview guide for adolescents explored initiation and progression of drug use, reasons behind use, and the impact of usage (see S1 Text for complete guide). Healthcare providers were asked about their perceptions on adolescent drug use, common drug-related issues, primary factors that contributed to adolescent usage, and perceptions of drug use among fellow healthcare providers (see S2 Text for complete guide). Both participant groups were asked about their perception of prevalence of specific drugs, trends of usage, and family reactions to adolescent drug use. Data collection was stopped once data saturation was reached.

### Analysis

Two researchers (BB and BZ) coded interview data using NVivo 12 and a thematic analysis approach, a widely used methodology for identifying shared meanings and patterns across a diverse set of experiences [30, 31]. Themes were identified by examining coded data in relation to the research objectives. By using Kleinman’s Eight Questions (e.g. “What do you think your sickness does to you?”), we developed a list of expected themes in our initial codebook (e.g. “perceived impact of drug usage”) [22]. After reviewing 16 interview transcripts, we inductively generated new codes to capture additional themes in raw data, leading to a finalized codebook that was used to code all interviews. Data from interviews with adolescent users and healthcare providers were triangulated to identify patterns and understand areas of convergence and divergence.

### Ethical issues

The Sangath Institutional Review Board approved the study (reference number: AN_2019_51). Written voluntary informed consent was taken individually from all participants. In the process of informed consent, individuals were provided with detailed information on the study’s objectives, the voluntary and confidential nature of their involvement, as well as its associated risks and benefits. Prior to enrolment, participants provided written consent after receiving comprehensive information. The study adhered to stringent protocols for confidentiality, data security, and management.

## Results

The 23 adolescents (mean age: 21.45 years [SD 1.9]) were seeking care for illicit drug use disorders at residential de-addiction centres (n = 9), non-governmental rehabilitation centres (n = 13), and public psychiatric care centres (n = 1). 12 adolescents were recruited from Delhi, 6 from Kerala, and 5 from Goa. 20 adolescents were males and 3 were females. 52% of the adolescents had schooling until the secondary level. 12 adolescents (52%) were employed and their monthly income ranged from Rs.1200 to Rs.10,000 [15–120 USD]. All adolescents reported previous use of tobacco, and 22 (96%) reported alcohol and cannabis use. 13 (56%) reported experience with depressants or sedatives, 12 (52%) with amphetamines, 9 (39%) each with inhalants and hallucinogens, and 8 (35%) with opioids.

The healthcare providers (n = 25) were psychiatrists (n = 3), clinical psychologists (n = 2), counselling psychologists (n = 5), nurses (n = 4), social workers (n = 8), physicians (n = 2) and program coordinators (n = 1). Seven healthcare providers were recruited from Delhi, seven from Kerala, and 11 from Goa. 10 providers were females, and the rest were males. They were associated with not-for-profit rehabilitation centres (n = 12), public healthcare centres (n = 5), and private healthcare clinics (n = 8).

**Fig 1:**
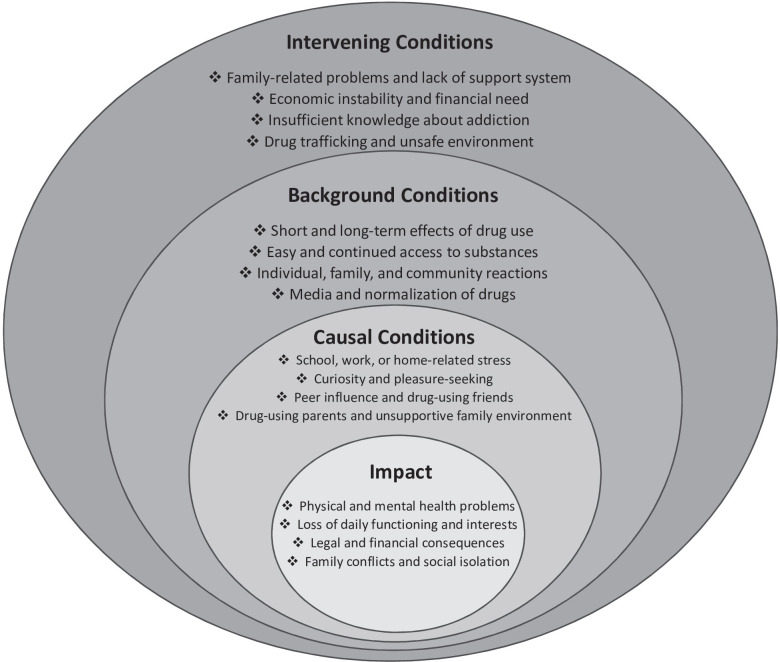
Explanatory model of illicit drug use in adolescents.

The themes we distilled from the interviews are reported below under four categories: a) general characteristics of drug use, b) initiation and progression of use, c) causal beliefs and d) perceived impact of drug use. A summary of the EM for adolescents’ experiences of illicit drug use is presented in Fig 1.

### A. General characteristics of drug use

When asked about their experiences with using legal as well as illegal substances, participants described their patterns of drug use and substance acquisition methods. They described the types of drugs used, frequency of usage, and mechanisms of drug acquisition (e.g. source of drugs and source of money to purchase drugs). Findings are reported below across two sub-themes.

### 1. Type and frequency of drug use

Adolescent participants commonly reported using multiple drugs concurrently. While they primarily used one or two drugs on a daily basis, other drugs were parallelly used on a weekly or monthly frequency: *“Cocaine*, *twice a week*. *Marijuana*, *daily*. *MDMA once a month*. *LSD*, *like once a month”* (Adolescent, Male, 24 years). One participant mentioned using smack (i.e., heroin) thrice a day, but most reported using a variety of substances at different times of the day. They described their days and nights as segmented by their drug use; *“Morning I would use alcohol and cannabis*. *Day time*, *I used opioids*. *In evenings*, *I used inhalants and at night*, *black charas”* (Adolescent, Male, 18 years).

Adolescent participants reported starting their day using substances, before proceeding with daily activities like taking a shower or having breakfast. One participant described how intertwined his daily schedule was with substance use: *“Right from morning I was doing this*. *Sometimes I won’t even brush in the morning*. *I would be sitting and smoking”* (Adolescent, Male, 21 years). Other participants described similar schedules that were structured around drug use. One of them shared how this was taking precedence over basic things like having meals: “*I stopped eating food and started using this more than food”* (Adolescent, Male, 21 years). One participant said that there was no real routine associated with drug use because it spanned his entire day. “*There was no limit for it*. *Smack*… *day and night*. *There was no time to sleep as well*. *Had to do it full night*. *There was no time-table*” (Adolescent, Male, 22 years). Another participant stressed the perpetual preoccupation with substance use that he experienced, and the endless, repetitive cycle of trying to find and use drugs every day. “*Every time I wake up*, *the first thing on my mind is*, *“How will I have my substance*? *How will I take my drug*? *What will I do today*?*” I would get my drugs*, *fix myself*, *and again think of another way to get it again*. *I would be out all day*, *thinking and trying to get the drug*, *my substance*. *Late night I come home*. *Sometimes I don’t sleep at all*. *Next morning*, *I will do the same*, *over*, *and over”* (Adolescent, Male, 24 years).

### 2. Mechanisms for drug acquisition

Some adolescent participants who were using Nitrosun (Nitrazepam) tablets had a prescription and acquired them from pharmacies. Participants also acquired “whiteners” from local stationary shops, using them as inhalants. Others acquired substances like smack, LSD, or cannabis from friends or fellow users. Healthcare providers highlighted how the circle of fellow users was essential to regular acquisition of drugs, since the financial burden of drug use was distributed among all the people in the circle. *“They buy it in turns*, *you know*. *If I spend 500 rupees on it*, *I invite over my friends*, *so next time it’s somebody else’s turn*.*”* (Provider, Male, 46 years).

Some adolescent participants who shared their source of acquisition mentioned a dealer. One of them added that the dealer would initially offer substances for free, before charging for subsequent transactions once they felt the need of using it more frequently. Another participant described in detail how they started with using drugs free of cost in a friend circle but eventually got involved in dealing with it. “*He used to give us samples for free*. *For free he used to give us different types of ganja*. *We tried everything*, *every kind of it*. *And he would tell us that you will get it for free if you sell this much and all*.*”* (Adolescent, Male, 21 years).

Health providers described an intricate network of drug users and dealers who were embedded in communities. They also mentioned that users and suppliers shared mutual knowledge about designated spots where transactions took place. Adolescent participants added that these “hotspots” could be found anywhere and everywhere in the cities. One of them, who had decided to move out of his city temporarily to be away from drugs, added context to this point. *“There are no places where you don’t get drugs here (in Delhi)*, *I don’t know about the small towns and the cities”*. (Adolescent, Male, 24 years).

Begging, stealing, drug dealing, and sex work were common sources of money to purchase substances for users who were unemployed and/or from low-income families. This issue was particularly pronounced among female users, who often lacked financial autonomy within their families or communities. “*She would use it maybe 5 times each day*, *each dose costing 500 INR*. *She would get the money by lying*, *stealing*, *begging*, *and other means to get money to buy the drugs”* (Translator for Adolescent, Female, 20 years). Some participants specifically mentioned stealing from family members.

A health provider added that female patients, especially from vulnerable backgrounds, often began dealing drugs as a source of income but eventually became users. This was in stark contrast to adolescent male users from middle- or high-income families who typically went into dealing drugs as a means to support their drug use. *“A dose of smack is around 300–350 INR*. *Girls might start as peddlers and then gradually become users*, *or they might get the money for drugs through sex work”* (Provider, Female, 43 years).

Overall, adolescent drug use seemed to be characterized by concurrent use of multiple substances, daily routines structured around drug consumption, and an endless cycle of seeking and using drugs. Adolescent participants acquired drugs from various sources including pharmacies, friend circles, and dealers and often resorted to stealing, drug dealing, or sex work to fund drug use.

### B. Initiation and progression of drug use

All adolescent participants reported first having tried legal or licit substances (alcohol or tobacco), before progressing to cheaper illegal/illicit substances (inhalants and cannabis), and then more expensive illicit substances (e.g., opioids, hallucinogens). This finding was confirmed by multiple health providers who shared that the trajectory of substance use in adolescents commonly started with easily acquired substances. *“The path of addiction is usually tobacco*, *then alcohol*, *then cannabis*, *and then opioids*.*”* (Provider, Female, 43 years).

Adolescent participants generally reported having used alcohol or tobacco for the first time between the ages of 12–16 years. The reason for first use was commonly reported as curiosity or a desire to *try*: *“I had a curiosity to know what it* [drinking] *was… After getting that feel*, *there was a feeling of need to have more*.*”* (Adolescent, Male, 24 years). Participants described their initial feelings of chewing tobacco or consuming alcohol as unpleasant, and reported feeling dizzy, uneasy and disoriented.

Cannabis and inhalants were the first illicit substances used by most adolescents, typically in the age of 13–16 years. Adolescent participants generally reported first using it in company of others, before starting its use alone. Almost all of them began using these substances out of curiosity or peer influence. One participant traced their initial cannabis usage to poor exam performance: *“I failed in school*. *Because of which I started taking weed*. *I used to only smoke it*. *I would forget all the tension that was there in my mind… I would only sleep after taking weed at night”* (Adolescent, Male, 21 years). Participants described the first experience of using MDMA and LSD as energetic and pleasurable. “*You’ll get energetic and you’ll be sober*. *You’ll think that you know everything*. *But the next day you get the bad trip—you go*” (Adolescent, Male, 21 years). They associated cannabis with relaxation and peace. Some participants felt inhalants made them aggressive and violent.

The continued use of substances like alcohol or cannabis despite the unpleasant first experience was not perceived as harmful by the adolescents since they did not *feel intoxicated*. The prevalent understanding was that usage was safe within limits that do not *make you high*. “*I wouldn’t get intoxicated drinking beer*. *So gradually I started sitting with them (his friends)*, *drinking alcohol*. *And I did not even realize that I got addicted to this thing*” (Adolescent, Male, 20 years). Some participants also reported using more often, only to experience the relaxation and light-headedness that their peers had described. *“Cannabis*, *I used it continuous for 15 days*, *and I was not getting any high or something like that*. *But after using it continuously*, *I started getting it*. *I liked it*, *it was different from alcohol”* (Adolescent, Male, 21 years).

In the experience of some participants, the line between experimentation and dependence was not very clear. The rapid escalation from occasional use to regular use, culminating in an inability to abstain, was difficult to distinguish as a problem on one’s own. “*I told my friend that I want to try it (cannabis)*. *Nothing happened to me that day*. *So then whenever I asked him*, *he gave me*. *He continued giving me and I continued taking it*. *I tried to quit it but I couldn’t*. *I couldn’t get sleep at night*. *I used to get restless*” (Adolescent, Male, 24 years).

To summarize, adolescents typically begin substance use with legal substances like alcohol and tobacco, driven by curiosity or peer influence, before progressing to cheaper illicit substances such as inhalants and cannabis, and eventually to more expensive ones like opioids. Despite unpleasant initial experiences, continued use was often perceived as harmless, leading to rapid escalation and difficulty distinguishing between experimentation and dependence.

### C. Causal beliefs

Participants shared the reasons they believe to have led them to initiate use or continue using. Five general sub-themes arose as causal attributions for illicit substance usage: peer influence, curiosity or pleasure, coping with stress, family conditions, and systemic risk factors.

### 1. Peer influence

Participants attributed frequent and progressive use of drugs to peer influence and shared use in friend circles. Engaging in drug use was perceived as crucial for inclusion and assimilation in peer groups. “*They also feel that if they don’t do* [substances] *then his friends will not value him… If they are also using*, *then they are like a team and are close to each other”* (Translator for Adolescent, Male, 24 years). Healthcare providers acknowledged that peer acceptance was formative for adolescents’ identity development, which may increase their susceptibility to peer-influenced drug use. Providers emphasized that friendships were prioritized over family and provided a feeling of connection and fraternity.

Adolescent participants also shared that after using drugs regularly for a while, they began consciously choosing friend circles where drugs were used and keeping in touch with only peers who found drug use acceptable. When asked about why taking drugs with your friends was important, one participant responded that it gave them a sense of support and feeling of belonging. *“It would give me courage*. *After getting intoxicated*, *it felt better to have someone to share your feelings with”* (Adolescent, Male, 23 years).

### 2. Curiosity and enjoyment

Curiosity was a major driving force for experimentation with illicit substances. Several participants pointed to drug use as a source of recreation and escape from boredom. One provider stated, *“There is nothing else to experiment with*, *right*? *In the cities*, *it’s like you have a few teen friends*, *and there is not even a playground near you… Often it comes to drugs”* (Provider, Male, 30 years). After initiation of substance use, pleasure seeking became the primary motivation to continue. Many adolescent participants reported experiencing both a curiosity to experiment and encouragement from peers to do so. “*I had the curiosity and peer pressure to take that kind of drug*. *The first time I took it*, *I fell in love with it*. *I told myself I’d never quit this drug*.*”* (Adolescent, Male, 24 years).

### 3. Coping with stress and managing emotions

Participants recounted that stress from school, work, or life obligations contributed to a need for relief and self-medication. One adolescent participant shared that he used drugs when he was depressed to forget the circumstances around him and feel better. *“I used to forget those things*. *I used to feel happy”*. (Adolescent, Male, 24 years). A provider supported this finding by conceptualizing substance use as a coping mechanism for adolescents. They noted that without awareness of how to handle situations and regulate emotions, adolescents frequently turn to drugs to manage their feelings; “*The primary cause would be the stress they are facing*, *and they don’t know how to overcome the stress”* (Provider, Female, 28 years). Some adolescents who started using only for pleasure, eventually used substances to cope with emotional distress caused by relationships or problems with friendships. *“He started off using it just for enjoyment and*, *and after that he was in depression*. *He would take drugs to get relief*” (Translator for Adolescent, Male, 23 years).

Other perceived factors included lack of support systems, chronic feeling of loneliness, stigmatization of drug users, and prevailing hopelessness and societal pessimism towards drug users. *“Society will not care much*. *They will always see us as a burden*. *Even if we are trying to do good*, *they will see us as enemies”* (Adolescent, Male, 24 years). Some adolescent participants shared that they eventually internalized these negative societal responses attached to prolonged drug use, making them feel underconfident and apathetic about the trajectory of their lives. One adolescent participant responded to this perceived “weakness” with further drug use: “*It would be like after taking drugs I used to feel like I can do something*. *I used to feel weak otherwise*.” (Adolescent, Male, 21 years).

### 4. Family conditions

Several adolescent participants associated their substance use with family-related conflicts and feelings of resentment towards parents. One participant mentioned that drug use made it easier to bear physical abuse inflicted by a parent. *“I used ganja*. *The reason was*, *I could be peaceful*. *Whatever my parents say*, *I would not care*, *even if they beat*, *I could not feel anything*. *Even if I get hurt*, *I would not feel the pain”* (Adolescent, Male, 21 years).

Multiple healthcare providers noted that having a parent with a substance use disorder increased the risk of the child using substances, particularly given economic instability. *“Economic stability is not there*. *Most of their fathers are having alcohol*. *Fathers never take care of their children*. *Even mothers are busy with their work”* (Provider, Female, 28 years). According to another provider, recovery was also deeply connected with one’s family conditions. *“Relapse may also occur when adolescents return to poor family conditions”* (Provider, Male, 34 years).

One participant shared that they started using inhalants to cope with a parent’s death when they were ten, but gradually observed that they felt like using it despite having no “good reason to use as such”. Another female participant, who grew up in a family of rag pickers in a slum in Delhi, shared that she ran away from home to a friend’s place, after suffering from prolonged verbal and physical abuse from her parents. While staying at the friend’s place, she started using drugs when offered because she could not refuse. *“It was her best friend*, *so she couldn’t say no*” (Translator for Adolescent, Female, 20 years).

### 5. Systemic and class-related risk factors

Both adolescent participants and providers highlighted that financial difficulties played a pivotal role in driving entry into cycles of drug dealing and illicit drug use. A provider emphasized how motivations for drug use were stratified by class, underscoring drug dealing as a means to achieve social mobility. *“Most people who are coming in as clients are either from the low socio-economic strata*, *or from the very high socio-economic strata*. *That low socio-economic strata… It’s a huge economic factor*. *So one of the client’s we had… His mother was bedridden*, *and the father left… So*, *he started peddling when he was in 9*^*th*^ *and all*, *because he had—uh hunger was immediate… And he was the only male so he started peddling and slowly he started using”* (Provider, Male, 28 years). One provider attributed adolescent substance use to the combination of socioeconomic stressors, lack of family supervision, insufficient knowledge about addiction, and easy accessibility to substances. Two health providers added that systemic factors like drug trafficking networks, “mafias”, and political-economic corruption also affect adolescents.

To summarize, adolescent substance use seems to be significantly influenced by peer pressure, with participants feeling that drug use is essential for social acceptance and connection within their friend groups. Additionally, factors such as curiosity, emotional coping, family conflicts, and socioeconomic challenges contribute to drug use, with many participants turning to substances as a means of escape or relief from stress and negative family dynamics.

### D. Perceived impact of drug use

Participants shared how drug use affected their personal, social and professional lives, while healthcare providers shared their learnings regarding the impact of drug use on their adolescent patients’ lives. Our findings show how drug use may impact adolescents’ health, everyday functioning, and relationships.

### 1. Health and daily functioning

Adolescent participants noted profound physical and mental impacts from extended substance use. Weight loss, diminished appetite, and persistent fatigue were highlighted. Adolescent participants felt that their stamina reduced and they were not able to adequately contribute to activities of their interest or academics. One participant stated, *“I was destroying myself*. *My body is not what it used to be*. *My performance and all*, *my brain wasn’t good as it was before 5 years ago”* (Adolescent, Male, 24 years). Others reported decreased stamina, confidence, and self-esteem. *“When I go for programs and all*, *I don’t go for family functions*. *I didn’t like to face them*. *Lack of confidence*, *depression*. *I feel I couldn’t reach where I should have*. *My talents were not utilized well*.*”* (Adolescent, Male, 21 years). Participants also reported struggling financially after having *wasted* a significant amount of money on substances.

Several adolescent participants described how compulsions for substances overwhelmed everyday functioning. A student recalled dropping out of school because drug use impacted his concentration. Another shared that he found it hard to retain things in his memory, which impacted his academic performance. “*My memory was diminishing*. *I could not even remember yesterday’s things”* (Adolescent, Male, 22 years). Users often isolated themselves and lost interest in activities that they previously found pleasurable. Another participant explained that they planned their day around drug use, which compromised everything else, including work: *"Every day I used to wake up and get ready for work*. *I used to think*, *I will smoke one joint and go to that shop*. *But when I have smoked one joint*, *I would not stop there only*. *I would smoke whole day*. *And not go to that shop"* (Adolescent, Male, 19 years).

### 2. Relationships with others

Adolescent participants also reported feeling irritable and angry with family members. For some of them, negative behavioral changes (e.g., expression of frustration) strained family relationships, while others mentioned a total breakdown of communication. *“I used to get angry on my mom when she was not giving me money*. *After that she was like*, *my son*, *I will give him because he is asking”* (Adolescent, Male, 20 years). Several adolescent participants reported a lack of interest in connecting with loved ones. “*I stopped talking to my dad*. *When my father used to call me*, *he used to tell me*, *"Tell something*, *speak something*.*" So I used to think*, *"What to say*, *what to speak*?*"”* (Adolescent, Male, 24 years).

Physical and emotional abuse by parents emerged as a common experience for adolescent participants when their drug use was discovered by family: *"Her family wouldn’t want her in the house*. *They used to tell her that they would cut her up and dump her into the river if she kept using”* (Translator for Adolescent, Female, 20 years). Healthcare providers described various emotions felt by families of affected adolescents: “*There’s a whole range of reactions… from denial to anger to acceptance*. *Some are very willing to seek treatment*. *Some don’t seek medical treatment*. *Some prefer more traditional forms of treatment… the local church*, *or those kind of religious interventions*, *traditional faith healers*. *They don’t see drug use a medical problem*. *They see it as a weakness of personality*, *or lack of faith in God”* (Provider, Male, 43 years).

One adolescent participant shared that he only retained relationships with other drug users because he felt socially included in their company and excluded elsewhere due to community stigma and misunderstanding. Adolescent participants felt that their reputation in their neighborhoods and society was affected. Several participants reported feeling that their social identity was reduced to their illness. *“Outside in society*, *my name was*, *bad*. *If someone come from outside and if they ask*, *"Do you know [participant name]*?*" People said*, *"Yeah*, *he is a druggie*.*"”* (Adolescent, Male, 24 years).

To summarize, adolescent participants reported significant physical and mental health consequences of drug use that further impacted their daily functioning, relationships, and self-esteem. Many participants reported strained family dynamics, social isolation, and a negative reputation in their communities due to their drug use.

Overall, our findings present a preliminary yet holistic picture of adolescent experiences with illicit drug use, summarized in the social-ecological model below (Fig 1). At its core, illicit drug use is characterized by its direct physical, mental, social, legal, and financial consequences upon an adolescent’s everyday life. Peer influence, drug-using parents, curiosity or pleasure-seeking, and psychosocial stress are perceived as direct factors contributing to an adolescent’s initial and continued use of illicit drugs. In addition to these causal elements, the societal normalization of drugs (e.g. media narratives glorifying drug use), proximity to “hotspots” with easy access to drugs, and family or community reactions that stigmatize drug use further isolated adolescent participants, perpetuating substance usage and preventing adolescents from seeking help. Finally, familial, social, and economic conditions may intervene at any point to further entrench adolescents in substance use; for instance, family or financial instability can incentivize an unemployed adolescent to begin dealing, and subsequently using, substances as a source of income, or it may encourage an adolescent from an abusive family to continue using drugs as a coping mechanism.

## Discussion

This qualitative study describes explanatory models of illicit drug use for adolescents in India. Nearly all adolescent participants reported previously using cannabis with depressants, inhalants, amphetamines, hallucinogens, and opioids. Usage of the latter substances progressed from initial experiences with readily available substances like tobacco, alcohol, and cannabis. Adolescent participants typically acquired illicit substances from pharmacies, fellow users, and drug dealers, embedded in an intricate network involving selling drugs or stealing money to purchase substances. All these findings are consistent with previous research from India [32–37].

Both providers and patients acknowledged the synthesis between systemic risk factors, family conditions, peer influence, and personal stress as driving forces of substance use. Almost all adolescent participants reported initial experimentation motivated by curiosity or peer influence. Continued drug use was normalized by media, peers, and family members, while also motivated by boredom, pleasure-seeking, school/work-related stress, and psychosocial causes. Structural inequalities disproportionately impact adolescents with low socioeconomic status (SES) due to lack of family support, stable income, and alternative healthy activities. Only providers referenced a biomedical model to characterize illicit substance use, while adolescents invoked personal and psychosocial explanations. The ripple-effect of illicit substances on adolescents’ physical and mental state, legal and family-related interactions, and everyday functioning suggests a pernicious cycle, where adolescents from disadvantaged backgrounds are more likely to engage in substance use and suffer harsher consequences [38].

This is the first study in India to construct an explanatory model of the mechanisms and social context in which adolescents acquire illicit substances, thus clarifying the disproportionate vulnerability of adolescents with low SES to become entangled in the network of drug use. Several studies have reported a quantitative association between low SES and adolescent drug use in India [34, 39], and our findings contribute to more nuanced understandings of the mechanisms underpinning this relationship. Specifically, we observe several processes: (i) adolescents with low SES may rely on drug dealing for income, increasing the likelihood of sustained substance use; (ii) families facing economic instability are less equipped to provide sufficient support, supervision, and healthy parental models for adolescents; (iii) adolescents from disadvantaged backgrounds experience greater stressors with less outlets for healthy activities, incentivizing usage of drugs for relief or pleasure; and (iv) adolescents from low SES may be more likely to encounter negative peer influences and drug networks. For female adolescents, these socioeconomic factors may be amplified by gender-based stigma and linkages between substance use and sex work. Our findings imply that treatment and prevention of substance use in Indian adolescents must account for the psychosocial and socioeconomic factors, as demonstrated by adolescents who relapse when returning to poor family conditions [40]. This suggests the need for comprehensive and targeted (e.g. gender-specific) interventions that address the psychosocial needs of adolescents, potentially involving financial assistance, individual and family counseling, community activities, and education-based prevention.

Our findings across North, West, and South India were internally consistent and externally reflect similar relationships reported in prior studies. Peer pressure, curiosity, and psychological stress have been observed as common reasons for initiating substance use [35, 41, 42]. Our finding that adolescents use substances to feel closer to their peers is consistent with studies that cite socialization and influence of peer groups as a substantial motive [43]. Emphasis on pleasure-seeking and recreation as reasons for continued substance use is reported elsewhere [34]. Studies of substance use amongst medical undergraduates in India describe foremost reasons for substance use as psychological stress, followed by pleasure-seeking [44, 45]. Unlike other studies, our sample did not explicitly mention easy availability of substances as a causal attribution of usage, but often highlighted the role of tension at home or school [43]. In addition, attribution of substance use to societal expectations and the popularity of drugs in media aligns with another study, which showed over 40% of adolescent substance users adopted the habit by watching celebrities using these products [39]. We also found relationships between SES and illicit drug use consistent with previous research; substance use is more common for students in families with less educated parents and lower SES, but adolescents from higher SES may have more pocket money that leads to greater risk-taking behavior and ability to acquire substances [46, 47].

Previous studies in India have reported individual factors like family income and peer influence affecting adolescent substance use through statistical models [48–50], but we could not find any comparable explanatory models across multiple regions. While there are limited qualitative studies of adolescent substance use in India, several authors also indicate peer influence, family dysfunction, and social norms as key factors for addiction [51–53]. Our findings on the impact of illicit substance use on adolescents’ everyday life are consistent with previous research in India that indicate similar challenges in school, employment, family and peer relations, legal problems, and psychiatric conditions [54]. Other studies have replicated similar findings, reporting difficulties faced by adolescent substance users regarding physical and mental health, poor academic performance, and family conflict [55, 56]. This explanatory model may support the effectiveness of culturally-adapted interventions in accounting for local contexts, illness models, and indigenous practices that influence acceptable and effective treatment [26, 57, 58].

There are some limitations to our study. First, interviews were carried out with adolescents currently in treatment for substance use, which represents a relatively small proportion of adolescents using substances in the population. Second, most of our participants were male. While there is a higher male adolescent substance users in India [42], greater stigma around drugs for females may discourage reporting and present disproportionate barriers to treatment [59]. Further studies are needed to investigate how gender mediates illicit drug use amongst Indian adolescents. Third, explanatory models may change based on different sociocultural factors, so our results may not be generalizable throughout India [39]. Fourth, although most of our interviews did not utilize an interpreter, the interpreters who assisted with interviews were of the same facility as adolescent participants, which may have biased the data collected. Additionally, it is possible the results are influenced by the researchers’ positionality, given the Asian-American backgrounds of KW and BZ. Fifth, data collection was conducted before the COVID-19 pandemic. The pandemic and social isolation may have affected various factors around adolescent substance use, including greater unemployment, family conflicts, and barriers to care [60, 61], but these changes follow the same causal relationships in our explanatory model.

## Conclusion

The evidence presented here is a first attempt to synthesize the direct influence of microsocial (e.g., family and school-related stress) and macrosocial (e.g., poverty and drug-trafficking) factors upon illicit drug use. Our study synthesizes themes from both adolescent participants and providers, compared to most studies on adolescent drug use in India that focus on just one perspective. A greater understanding of how adolescent participants and their providers conceptualize initiation of substance use, the resulting impact, and the factors that influence continued usage may ground future negotiations between patient and clinician. Such an explanatory model has the potential to enhance patient-provider relations, compliance to treatment, and intervention outcomes.

The crucial role of stress and peer influence on initiation and continuation of substance use amongst adolescent participants highlights the need for effective interventions, like school-based prevention and adolescent ambassador programs that leverage peer outreach [62, 63]. The connections between adolescent drug usage and family-related stressors also suggest the necessity of family therapy, which has been more effective than other treatments in retention and reducing adolescent drug use [64]. As socioeconomic challenges are key contributors to the initiation and relapse of illicit drug use, specific approaches targeting these difficulties (e.g. problem-solving strategies and financial assistance for patients) may be necessary for effective treatment, beyond government schemes focused on education and demand reduction around drugs [65]. Thus, this study has important implications for intervention development and clinical treatment of adolescent substance use in India.

## Supporting information

S1 File COREQ checklist. (PDF)

S1 Text Interview guide for adolescents. (DOCX)

S2 Text Interview guide for healthcare providers. (DOCX)
