# Supplementary material for: Explanatory models of illicit drug use in adolescents: A qualitative study from India
Source: PLOS Glob Public Health. 2024 Oct 14;4(10):e0003647. doi: 10.1371/journal.pgph.0003647 (PMC11472909; doi:10.1371/journal.pgph.0003647)
Supplement: S1 Text — (DOCX) [file pgph.0003647.s002.docx]

A Qualitative Assessment of Adolescent Drug Use in India

Script & Qualitative Interview Guide

**SECTION 1: Introduction**

Hello! My name is ____, and I am a researcher affiliated with the mental health NGO Sangath. I am working on a study to understand illicit drug use in adolescents. (In this study, adolescents refer to young people ages 18-24.) The goal of this project is to learn more about the characteristics of usage in this group, the factors that contribute to it, and how people handle their usage. We will be interviewing people aged 18-24 across different Sangath projects and partner organizations, as well as some healthcare providers. I’d like to ask you some questions about your personal experience with using.

**Interviewer Checklist**

Bring:

- Audio recorder
- Cue cards
- Socio-demographic form
- Tablet
- Consent/info form
- Pen/paper
- Incentive

Mention:

- Your name
- Goal of the project
- Consent form
- Confidentiality – ***emphasise this***
- Speak openly and honestly
- Socio-demographic form
- Use of audio recorder

But first, I need your consent. This sheet outlines the purpose of the study, your rights as a participant, and people you can contact if you have concerns. Let’s review the main points.

[REVIEW KEY POINTS IN INFORMED CONSENT FORM MAIN TAKEAWAYS DOCUMENT WITH PARTICIPANT.]

One point is recording. If it’s ok with you, I’d like to record our conversation to make sure my analysis is accurate later. Only my research team and I will listen to these recordings. *(I will only share them as anonymous comments.)* May I record our conversation?

[PAUSE]

Once you’re done reading this, you can ask me any question, then sign it.

Before we begin, I will be asking you basic demographic questions and putting your answers on this tablet. I will not put your name down, just your participant ID. Then, we will start the interview. This is a judgement-free zone. I will mostly be listening to you, but I may ask you to explain your responses. I will be taking some notes to help me track our conversation. This interview should last 1 hour.

Please remember that everything you share is confidential, so without your permission, we will not share anything that can be linked back to you. Only anonymous data may be shared with other researchers and in dissemination activities. *(This means that if we use any of your quotes, they will be anonymized, and nobody will be able to trace your comments to you.)* Please be honest and open with your comments! There are no right or wrong answers. Before we begin, do you have any questions about the study?

[PAUSE AND ANSWER QUESTIONS]

[PAUSE AND FILL OUT SOCIODEMOGRAPHIC FORM]

Let’s go ahead and get started with the interview. Please stop me if you want to take a break, or if you would like any part of the interview to not be recorded.

[TURN ON TAPE RECORDER]

**SECTION 2: Characteristics of Drug Usage – Individual Level**

First, I’d like to ask you some general questions about your personal drug use.

2.1. Think back to the first time you ever used any drug. This includes licit ones, like alcohol and tobacco, and illicit ones. If you remember, can you describe what happened?

***Interviewer Note: Hold up Card 1***

*For the purposes of this study, illicit drug refers to substances that are not medically prescribed and/or are illegal to produce and sell. These drugs include: hash, ganja, LSD (acid), psilocybin (shrooms), opium, heroin, MDMA, ketamine, cocaine (Charlie), etc. Licit drugs are legal and/or are medically prescribed.*

🡪 Probes:

- - Which drug were you using?
  - How old were you?
  - Who were you with?
  - Where did you get the drug?
  - How much did you take?
  - How did you feel before taking the drug?
  - How did you feel while you were taking the drug?
  - How did you feel after taking the drug?

*[IF FIRST DRUG USED WAS* ***LICIT*** *🡪 CONTINUE TO* ***2.2****]*

*[IF FIRST DRUG USED WAS* ***ILLICIT*** *🡪 SKIP TO* ***2.3****]*

- 1. Now, think about the first time you used an *illicit* drug. If you remember, tell me more about that experience.
  - Which drug were you using?
  - How old were you?
  - Who were you with?
  - Where did you get the drug?
  - How much did you take?
  - How did you feel before, during, and after taking the drug?
  1. In the past 12 months before your time at [ORGANISATION], which illicit substances have you used?

🡪 Probes:

- Are you currently using anything?
  1. Can you describe what a typical occasion might look like when you’re using [MENTIONED SUBSTANCES]?

🡪 Probes:

- - ***Interviewer Note****:* ***Please make sure to ask this probe*** *-* In the past 12 months before your time at [ORGANISATION], how often have you used [MENTIONED SUBSTANCES]?
  - Where do you usually consume the drugs?
  - When do you use each substance?
  - Where do you obtain the substances?
  - How do you take the drugs (eating, smoking, etc.)?

2.5. Can you tell me more about the reasons behind your use of [MENTIONED SUBSTANCE #1/#2/#3/ETC.]?

***Interviewer Note:***

*Make sure to ask this question for each drug mentioned.*

🡪 Probes:

- - What pressures do you feel to use?
  - What are factors in your life that contribute to your usage?
  - Who around you (friends, family) uses, if at all?
  - Which drug do you use the most? Why?

2.6 In what ways has using [MENTIONED SUBSTANCES] impacted you?

***Interviewer Note:***

*These impacts may be positive or negative. Examples of “impact” may include increased confidence, gaining or strengthening relationships, pain suppression, social inclusion, addiction, weight gain, depression, anxiety etc. It may also include missing school, getting lower grades, losing relationships, social exclusion, euphoria, etc.*

🡪 Probes:

- - How has it affected your physical and/or mental health?
  - How has it affected your relationships with others?
  - How has it affected your social functioning?
  - How has it affected your functioning at college/work?

**SECTION 3: Healthcare-Seeking Behaviours**

Now, let’s move onto some questions about how you and others might deal with frequency of using.

3.1. Before coming to [ORGANISATION], did you ever try to change the amount that you’re using? Why or why not?

***Interviewer Note:***

*“Change” may refer to an increase as well as a decrease in usage.*

🡪Probes:

- - What triggered it?
  - How do your attempts before treatment compare to your attempts during treatment?

[*IF YES, CONTINUE ONTO* ***3.2]***

*[IF NO, SKIP TO* ***3.3****]*

3.2. Please describe the ways you used to change your amount of use before coming to [ORGANISATION]. How did you try to change your use?

***Interviewer Note:***

*The participant’s answer to 3.1 may lead into 3.2, in which case it is not necessary to ask 3.2. However, do ask the probes within 3.2.*

*Examples of reduction strategies may include speaking to friends and family, meditating, journaling, self-medicating with legal drugs, faith, etc. They can also be medical in nature and include rehabilitation services, doctor consultations, acupuncture, different forms of therapy, support groups, etc.*

🡪Probes:

- - What is the strategy?
  - How often do you use the strategy or support?
  - How much did this strategy or support cost?
  - How did you find out about the strategy or support?
  - Why did you pick this strategy or support?
  - What were the results of using the strategy or support?
  - (IF MORE THAN ONE STRATEGY IS LISTED) Which strategies were the most effective? Least effective?

3.3. What caused you to seek services at [ORGANISATION]?

🡪Probes:

- - What were your motivations?
  - How did you feel about entering [ORGANISATON]?

3.4. At [ORGANISATION], what strategies or supports are you currently using to eliminate your usage?

🡪Probes:

- How has [ORGANISATION] been helping you with this strategy or support?
- How often do you use the strategy or support?
- What are the results of using the strategy or support?
- How effective do you find this strategy or support?

3.5. If/when you wanted to reduce usage, what resources are available to you and your peers in your community?

***Interviewer Note:***

*Examples of resources might include counselling, subsidized or free healthcare, religious services, peer networks, community centre resources, family support, etc.*

🡪Probes:

- How accessible are the resources?
- Would you ever use them? Why or why not?

3.6. If/when you wanted to reduce usage, what sort of resources would you like to have?

***Interviewer Note:***

*Examples of resources can include the following:*

- *Peer counseling or support groups*
- *Technology*
- *Social media campaign*
- *Community-based programs*
- *Family intervention therapy*
- *Education*
- *Psychosocial intervention*

🡪Probes:

- - When would you have liked to have these resources?

**SECTION 4: Characteristics of Drug Usage – Structural Level**

I’d like to end with some broader questions, moving away from your personal use. When I say “young people who use drugs,” I am referring to not just you but also the group as a whole. You may be including yourself when you answer, but I’d like you to think about people ages 18-24 *in general*.

- 1. Do you think illicit drug use is common in your community? Why or why not?

🡪 Probes:

- How would you summarise drug use in [STATE]?

5.2. What do you think are the primary factors that contribute to use among people your age in your community?

🡪Probes:

- - What role does the media play? Industrialization? Globalization?
  - What role does others’ expectations play?

5.3 How do people your age react to youth who use illicit drugs? How does your family react? Teachers? Other adults? Other members of you community?

***Interviewer Note:***

*This question refers to people who use with any frequency.*

🡪Probes:

- - How do those reactions differ among groups (peers, adults, teachers, family, etc.)?

**SECTION 5: Wrap-Up**

6.1. Is there anything else that you think is important and I have not asked?

Thank you so much for participating in this study. Over the next few months, we’ll summarise the information we’ve gathered from you and the other participants. What you have shared today will help us gain a better understanding of this important health behaviour so that we can better support adolescents in India and beyond. Thank you!

**General Probes**

Elaboration

- *Silence and eye-contact*
- Could you tell me more about that?
- Why do you think this?
- How so?
- Could you provide me with an example of this?
- What was that like for you?
- How did that make you feel?

Clarification

- Could you be more specific about this?
- What do you mean by that?
- *Echo – rephrase and summarize*

Redirection

- Thank you for sharing this. Let’s return to the primary question…
- Why don’t we move on to talk about [topic]?
- Let’s stay focused on [topic].
