# Supplementary material for: Explanatory models of illicit drug use in adolescents: A qualitative study from India
Source: PLOS Glob Public Health. 2024 Oct 14;4(10):e0003647. doi: 10.1371/journal.pgph.0003647 (PMC11472909; doi:10.1371/journal.pgph.0003647)
Supplement: S2 Text — (DOCX) [file pgph.0003647.s003.docx]

A Qualitative Assessment of Adolescent Drug Use in India

Script & Qualitative Interview Guide

**SECTION 1: Introduction**

**Interviewer Checklist**

Bring:

- Audio recorder
- Socio-demographic form
- Tablet
- Consent/info form
- Pen/paper
- Incentive

Mention:

- Your name
- Goal of the project
- Consent form
- Confidentiality – ***emphasise this***
- Speak openly and honestly
- Socio-demographic form
- Use of audio recorder

Hello! My name is ____, and I am a researcher affiliated with the mental health NGO Sangath. I am working on a study to understand illicit drug use in adolescents. (In this study, adolescents refer to young people ages 18-24.) The goal of this project is to learn more about the characteristics of usage in this group, the factors that contribute to it, and how people handle their usage. We will be interviewing healthcare providers across different Sangath projects and partner organizations, as well as people aged 18-24. I’d like to ask you some questions about your experience providing care for people in this age group who use illicit drugs.

But first, I need your consent. This sheet outlines the purpose of the study, your rights as a participant, and people you can contact if you have any concerns. Let’s review some of the main points.

[REVIEW KEY POINTS IN INFORMED CONSENT FORM MAIN TAKEAWAYS DOCUMENT WITH PARTICIPANT.]

One point is recording. If it’s ok with you, I’d like to record our conversation to make sure my analysis is accurate later. Only my research team and I will listen to these recordings. (*I will only share them as anonymous comments.)* May I record our conversation?

[PAUSE]

Once you’re done reading this, you can ask me any question, then sign it.

Before we begin, I will be asking you basic demographic questions and putting your answers on this tablet. I will not put your name down, just your participant ID. Then, we will start the interview. I will mostly be listening to you, but I may ask you to explain your responses. I will be taking some notes to help me track our conversation. This interview should last 1 hour.

Please remember that everything you share in our conversation is strictly confidential, meaning that without your permission, we will not publicly share anything that can be linked back to you. Only anonymous data may be shared with other researchers and in research dissemination activities. *(This means that if we use any of your quotes, they will be anonymized, and nobody will be able to trace your comments to you.)* Please be honest and open with your comments! There are no right or wrong answers. Before we begin, do you have any questions about the study?

[PAUSE AND ANSWER QUESTIONS]

[PAUSE AND FILL OUT SOCIODEMOGRAPHIC FORM]

Let’s go ahead and get started with the interview. Please stop me if you want to take a break, or if you would like any part of the interview to not be recorded.

[TURN ON TAPE RECORDER]

**SECTION 2: Characteristics of Drug Usage – Individual Level**

First, I’d like to ask you some general questions about the healthcare you provide.

2.1. To whom do you currently provide care?

2.2. For what sort of issues do your patients ages 18-24 commonly come to see you?

***Interviewer Note:***

*These issues may be not only medical but also social, emotional, etc. in nature.*

🡪Probes:

- - Why do they come in for your services?
  - What triggers them to come in for services?

2.3 What sort of drug-related issues do you encounter in your patients?

🡪Probes:

- - Out of the illicit drugs used, which ones are the most used?
  - What trends have you noticed in these issues over the years?

2.4. What forms of support do you provide to your patients?

***Interviewer Note:***

*Examples of support may include speaking to friends and family, meditating, journaling, self-medicating with legal drugs, faith, etc. Examples of support may also include rehabilitation services, doctor consultations, acupuncture, different forms of therapy, support groups, etc.*

🡪Probes:

- - How often do you provide each support?
  - How effective are these supports?
  - What sort of follow-up do you do with patients?

**SECTION 3: Characteristics of Drug Usage – Structural Level**

Now, I’d like to ask some broader questions on adolescent drug use. When I say “young people who use drugs,” I am referring to not just your patients but also the group of people aged 18-24 as a whole. You may be including your patients when you answer, but I’d like you to think about people ages 18-24 *in general*.

- 1. Do you think illicit drug use in adolescents is common in your community? Why or why not?

🡪Probes:

- What are your thoughts on drug use in [STATE]?
  1. What do you think are the primary factors that contribute to use among young people in your community?

🡪Probes:

- What role does the media play? Industrialisation? Globalisation?
- What role does others’ expectations play?
  1. How do your fellow providers react to young people who use illicit drugs? How do adolescents react? Family? Teachers? How about other members within your community?

🡪Probes:

- - How do those reactions differ among groups (your fellow providers, adolescents, teachers, family, etc.)?

**SECTION 4: Healthcare-Seeking Behaviors**

Here are some questions about forms of support for adolescents. “Support” refers to therapy, treatment, help, or care.

4.1. What do you think are barriers that prevent adolescents from seeking support to decrease their drug use?

4.2. What do you think are factors that help adolescents seek support to decrease their drug use?

4.3. In your opinion, what do you think would be an effective form of intervention to prevent and/or treat illicit drug use in adolescents?

***Interviewer Note:***

*Examples of interventions can include the following:*

- *Policy*
- *Peer counseling or support groups*
- *Technology*
- *Social media campaign*
- *Community-based programs*
- *Family intervention therapy*
- *Education*
- *Psychosocial intervention*

🡪Probes:

- - When should these interventions be implemented?

4.4. What do you think could be improved at the primary, secondary, and/or tertiary levels of care for this group?

***Interviewer Note:***

*If the participant has trouble with this question, you may rephrase it as “What do you wish could be improved at your level of care (tertiary) to provide better support for adolescents?”*

*The primary level of care refers to the first level of contact between patients and the healthcare system. This usually includes general practitioners, physicians, and physiotherapists, and the services typically provided are immunisation, basic healthcare, prevention of disease, etc. The secondary level of care refers to healthcare services that are provided by medical specialists who may not have primary contact with patients, often at district hospitals or community centers. This includes cardiologists, urologists, psychiatrists, etc. The tertiary level is specialized healthcare for patients referred by primary and secondary health professionals. Examples of services include cancer management, specialized surgeries, and advanced diagnostics at medical colleges or advanced medical research universities.*

🡪Probes:

- - Why is it important for this improvement to be made?
  - What would be the long- and short-term impacts of such improvements?
  - What other support should be made available at those levels that are not currently available?

**SECTION 5: Wrap-Up**

5.1. Is there anything else that you think is important that I have not asked?

Thank you so much for participating in this study. Over the next few months, we’ll summarize the information we’ve gathered from you and the other participants. What you have shared today will help us gain a better understanding of this important public health issue so that we can better support adolescents in India and beyond. Thank you!

**General Probes**

Elaboration

- *Silence and eye-contact*
- Could you tell me more about that?
- Why do you think this?
- How so?
- Could you provide me with an example of this?
- What was that like for you?
- How did that make you feel?

Clarification

- Could you be more specific about this?
- What do you mean by that?
- *Echo – rephrase and summarize*

Redirection

- Thank you for sharing this. Let’s return to the primary question…
- Why don’t we move on to talk about [topic]?
- Let’s stay focused on [topic].
